# Injuries With Electric vs Conventional Scooters and Bicycles

**DOI:** 10.1001/jamanetworkopen.2024.24131

**Published:** 2024-07-23

**Authors:** Adrian N. Fernandez, Kevin D. Li, Hiren V. Patel, Isabel Elaine Allen, Umar Ghaffar, Nizar Hakam, Benjamin N. Breyer

**Affiliations:** 1 Department of Urology, University of California, San Francisco; 2 Department of Epidemiology and Biostatistics, University of California, San Francisco

## Abstract

**Importance:**

Micromobility, the use of small vehicles (primarily scooters and bicycles), has become a standard transportation method in the US. Despite broad adoption of electric micromobility vehicles, there is a paucity of data regarding the injury profiles of these vehicles, particularly in the US.

**Objective:**

To characterize micromobility injury trends in the US, identify demographic characteristic differences in users of electric and conventional vehicles, and identify factors associated with hospitalization.

**Design, Setting, and Participants:**

This cross-sectional study queried the National Electronic Injury Surveillance System, a comprehensive database that collates injury data associated with consumer products from emergency departments across the US to provide national estimates, from calendar year 2017 to 2022. Data on micromobility vehicle injuries (bicycles, scooters, electric bicycles [e-bicycles], and electric scooters [e-scooters]) were obtained.

**Main Outcomes and Measures:**

Trends in injury and hospitalization counts, injury characteristics, and factors associated with hospitalization.

**Results:**

From 2017 to 2022, the US recorded 2 499 843 bicycle (95% CI, 1 948 539-3 051 147), 304 783 scooter (95% CI, 232 466-377 099), 45 586 e-bicycle (95% CI, 17 684-73 488), and 189 517 e-scooter (95% CI, 126 101-252 932) injuries. The median age of the riders was 28 (IQR, 12-51) years; 72% were male, 1.5% Asian, 13% Black, 12% Hispanic, and 49% White. Annual e-bicycle and e-scooter injuries increased from 751 (95% CI, 0-1586) to 23 493 (95% CI, 11 043-35 944) and injuries increased from 8566 (95% CI, 5522-11 611) to 56 847 (95% CI, 39 673-74 022). Compared with conventional vehicles, electric vehicle accidents involved older individuals (median age, 31 vs 27 years; *P* < .001) and a higher proportion of Black riders (25% vs 12%; *P* < .001). Helmet use was less in electric vehicle incidents compared with conventional vehicles (43% vs 52%; *P* = .02), and injuries were more common in urban settings (83% vs 71%; *P* = .008). Age-adjusted odds of hospitalization among all Black individuals compared with White individuals was 0.76 (95% CI, 0.59-0.98; *P* = .04).

**Conclusions and Relevance:**

In this cross-sectional study of micromobility vehicles, an increased number of injuries and hospitalizations was observed with electric vehicles compared with conventional vehicles from 2017 to 2022. These findings suggest the need for change in educational policies, infrastructure, and law to recenter on safety with the use of micromobility vehicles.

## Key Points

**Question:**

Are demographic characteristics, injury types, and health care use associated with injuries involving electric scooters or bicycles different from those involving conventional scooters or bicycles?

**Findings:**

In a cross-sectional study including 86 623 individuals, electric bicycle injuries increased by nearly 100% and electric scooter injuries increased by more than 45% annually. Injured electric vehicle users were older and less commonly helmeted than those injured from conventional vehicles, with significantly lower odds of hospitalization in individuals who were Black than in those who were White.

**Meaning:**

The findings from this study suggest that safer riding infrastructure and rider practices are important to curtail the rise of micromobility injuries.

## Teaser

This cross-sectional study examines the number of injuries and hospitalizations associated with electric vs conventional bicycles and scooters from 2017 to 2022.

## Introduction

Micromobility is the use of small vehicles, primarily electric and nonelectric (conventional) bicycles and scooters, designed for 1 or 2 passengers. Shareable scooter and bicycle platforms (eg, Lime Micromobility, Lyft Bikes, and Citi Bike) have become available in many major cities, appealing to consumer and urban planners interested in low-cost, low-emission, and readily available transportation.^1,2,3^ These small vehicles tackle the first and last mile challenge, allowing riders to get from home and work to centers of mass public transit without the use of an automobile, reducing emissions and congestion.^4^ Micromobility ridership in the US has increased more than 50-fold in the past 10 years, and the growing market is projected to value $300 billion in the US by 2030.^1,5^

Electric micromobility vehicles, such as electric scooters and bicycles (e-scooters and e-bicycles), were recently adopted by US vehicle sharing schemes and their use subsequently surged in popular use.^3,6,7,8^ With electric-powered motor assistance, e-scooter and e-bicycle riders can cover more distance with less effort, lowering transportation and exercise barriers for less-fit individuals.^9,10^ There are benefits—health, environment, traffic—to widespread electric micromobility use, yet there are concerns regarding injury risk to riders as well.^11^ Shared vehicle platforms rarely offer helmet rentals, and the fast acceleration of these heavier electric machines can prove dangerous, especially in the hands of novice renters.^12,13^

Despite the increasing popularity of micromobility in the US and the introduction of electric small vehicles, there is a paucity of data regarding the public health impact of these transportation sector changes.^1,14^ We hypothesized that electric vehicles (EVs) account for a growing percentage of micromobility-related injuries in the US and expect that demographic characteristics are distinct between EV and conventional riders. The present study characterized injuries and hospitalizations from e-bicycles and e-scooters compared with conventional bicycles and scooters across the US from calendar year 2017 to 2022. Ultimately, this work is intended to aid in the development of safer micromobility infrastructure and promote responsible riding practices.

## Methods

We followed the Strengthening the Reporting of Observational Studies in Epidemiology (STROBE) reporting guideline for reporting cross-sectional studies. Because all data were obtained from a publicly available database, this study did not constitute human research and does not require institutional review board review or exemption according to the Common Rule (45 CFR 20 §46).

### Data Source

**Figure 1.**
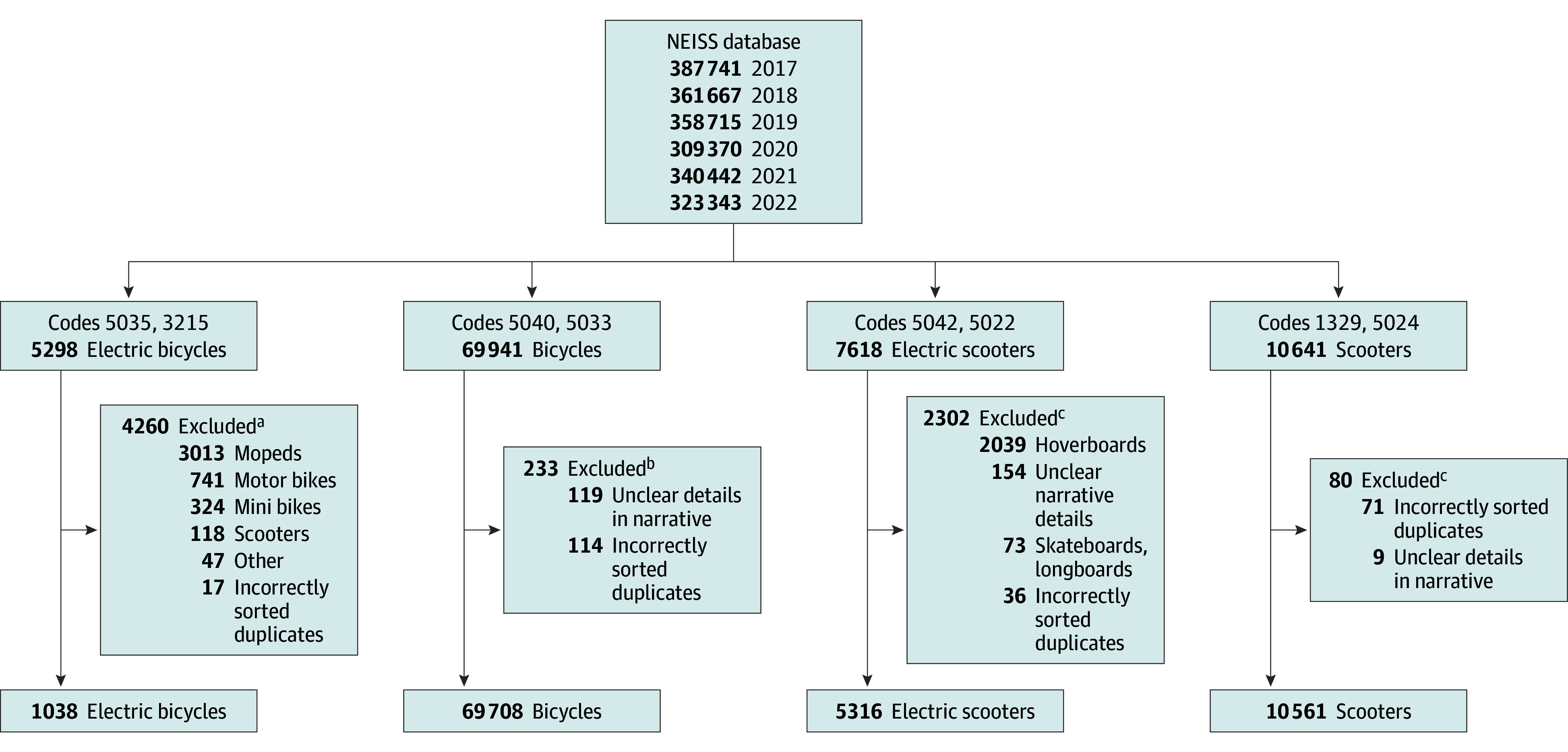
Creation of Vehicle Cohorts From the National Electronic Injury Surveillance System (NEISS) Database ^a^Narrative contains *electric bike* or variations. ^b^Narrative contains *bike*, *biking*, or variations. ^c^Narrative contains *scooter*.

We obtained data on micromobility vehicle injuries from the National Electronic Injury Surveillance System (NEISS), a comprehensive database managed by the US Consumer Product Safety Commission.^15^ The NEISS collates injury data associated with consumer products from approximately 100 emergency departments (EDs) across the US and its territories. The EDs were chosen through a stratified sampling process that includes different hospital sizes and children’s hospitals to ensure national representativeness. The database undergoes annual adjustments to its statistical weights to reflect the national distribution of ED visits accurately. Our study period covered 2017 to 2022, focusing on injuries involving e-bicycles, e-scooters, bicycles, and scooters. We identified relevant cases using NEISS product codes and injury narratives (Figure 1). In addition to injury categorization through medical records review at participating centers, the NEISS gathers information on each injury incident, including patient demographic characteristics (age, race and ethnicity, gender); injury location; diagnosis; body part injured; drug, alcohol, and fire involvement; products involved; injury disposition, and a short description of the injury event.

### Covariates and Outcomes

We included the following variables: race and ethnicity (American Indian, Asian, Black, Hispanic or Latino, Pacific Islander, White, and other [includes individuals identified in the NEISS database as African, Bengali, Central American, Dominican, Guatemalan, Honduran, Lithuanian, Mexican, Multiracial, Nepali, Puerto Rican, Russian, Somali, South American, Spanish, Turkish, and Ukrainian], as categorized in the NEISS), injury region (head or neck, trunk, upper extremity, and lower extremity), injury type (blunt, sharp [ie, lacerations or punctures], orthopedic/fracture, burn/shock, neurologic, internal, dental/facial, amputation/avulsion, other, and unknown), and hospital type (urban, rural, children’s). Race and ethnicity were included to characterize demographic characteristic changes among those injured in micromobility accidents during the stated time period and were coded based on the classifications recorded in the emergency department records at participating hospitals. Helmet use was determined using a text-search algorithm in injury narratives. Instances with *helm* preceded by *no*, *without*, *w/o*, or *unhelmeted* indicated no helmet was used. Narratives with *helm* but without the aforementioned qualifiers were presumed to indicate helmet usage. This algorithm was validated to be 95% accurate (300 of 316 correctly coded by manual narrative review in the e-bicycles cohort). To calculate helmet use statistics, only cases in which narratives explicitly referenced helmet use (17 935 of 86 623 injuries) were included. As a primary outcome, we identified injuries and hospital admissions (NEISS disposition: treated and transferred, treated and admitted/hospitalized, and held for observation) from each vehicle type over the stated time period.

### Statistical Analysis

National estimates and proportions of injuries and hospitalizations were derived using stratified, weighted, and nested estimates accounting for NEISS complex survey design, with 95% CIs calculated per NEISS guidelines.^15^ Injury estimates were plotted by year and age group with injuries leading to hospitalization distinctly colored. Continuous variables were analyzed with the survey-weighted Wilcoxon rank-sum test, and categorical variables were analyzed with survey-weighted χ^2^ tests with Rao and Scott second-order correction. Post hoc analyses were conducted with corrections for multiple comparisons using the Holm sequential correction method to control the family-wise error rate.^16^ To evaluate annual trends, we applied linear regression to log-transformed yearly injury estimates and analyzed the probability of hospitalization across years using survey-weighted logistic regression with year included as a covariate. To quantify associations with hospital admission, we created separate survey-weighted logistic regression models, each adjusted for age as a potential confounder and stratified by vehicle type. All analyses were conducted in R, version 4.3.1, statistical software using the survey package (R Project for Statistical Computing). A 2-sided value of *P* < .05 was considered significant and significance testing was unpaired.

## Results

**Figure 2.**
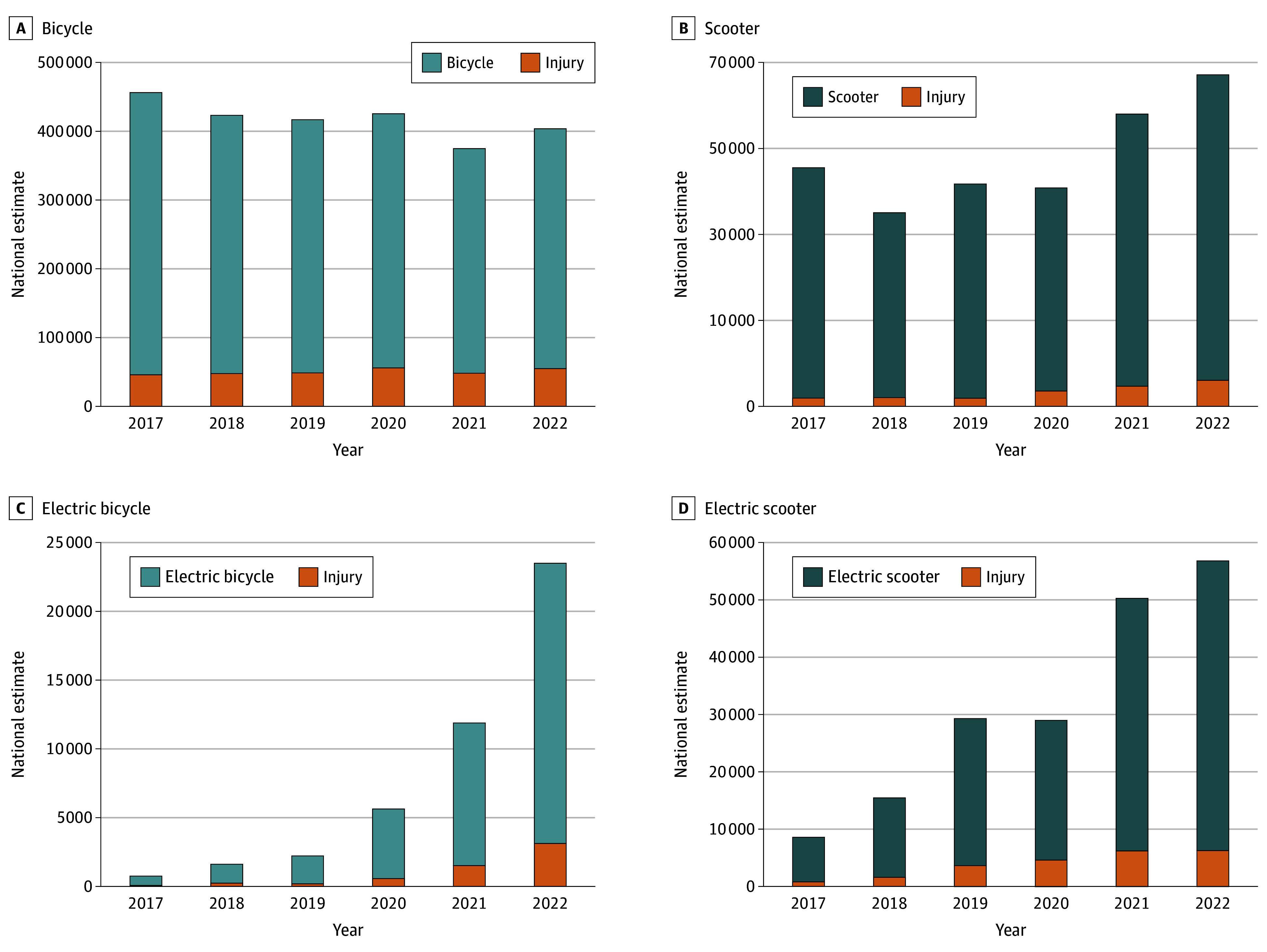
Estimated National Injuries and Hospitalizations per Year by Vehicle Type Injury indicates those resulting in hospitalization.

From 2017 to 2022, a weighted total of 2 499 843 bicycle (95% CI, 1 948 539-3 051 147 [69 708 NEISS cases]), 304 783 scooter (95% CI, 232 466-377 099 [10 561 NEISS cases]), 45 586 e-bicycle (95% CI, 17 684-73 488 [1038 NEISS cases]), and 189 517 e-scooter (95% CI, 126 101-252 932 [5316 NEISS cases]) injuries occurred in the US (Figure 1 and Figure 2). Injured riders (N = 86 623) had a median age of 28 (IQR, 12-51) years. Most were male (72% vs 28% female) and racial composition was 2% Asian, 13% Black, 49% White, and 3% other; 12% of the population identified as Hispanic.

### Micromobility Injury and Hospitalization Trends

Combined injuries across all vehicle types did not increase significantly from 2017 to 2022, although total hospitalizations across all vehicle types increased from approximately 48 863 (95% CI, 32 705-65 021) in 2017 to 70 644 (95% CI, 41 007-100 281) (*P* = .001) in 2022 (eTable 1 and eTable 3 in Supplement 1). e-Bicyclist injuries increased by more than 99% annually (*P* < .001), from 751 (95% CI, 0-1586) in 2017 to 23 493 (95% CI, 11 043-35 944) in 2022. e-Scooter injuries increased by more than 45% annually (*P* = .002), from 8566 (95% CI, 5522-11 611) to 56 847 (95% CI, 39 673-74 022) during this period (Figure 2; eTable 1 in Supplement 1). There was not a significant change in the number of injuries among bikers or scooter riders between 2017 and 2022 (eTable 1 in Supplement 1). Odds of hospitalization among injured EV users did not change substantially from 2017 to 2022, but increased significantly among conventional bicyclists annually by more than 7% (*P* = .003) and scooter riders by more than 17% (*P* < .001) (Figure 2; eTable 1 in Supplement 1).

### Rider and Hospital Demographic Characteristics

#### Age

**Figure 3.**
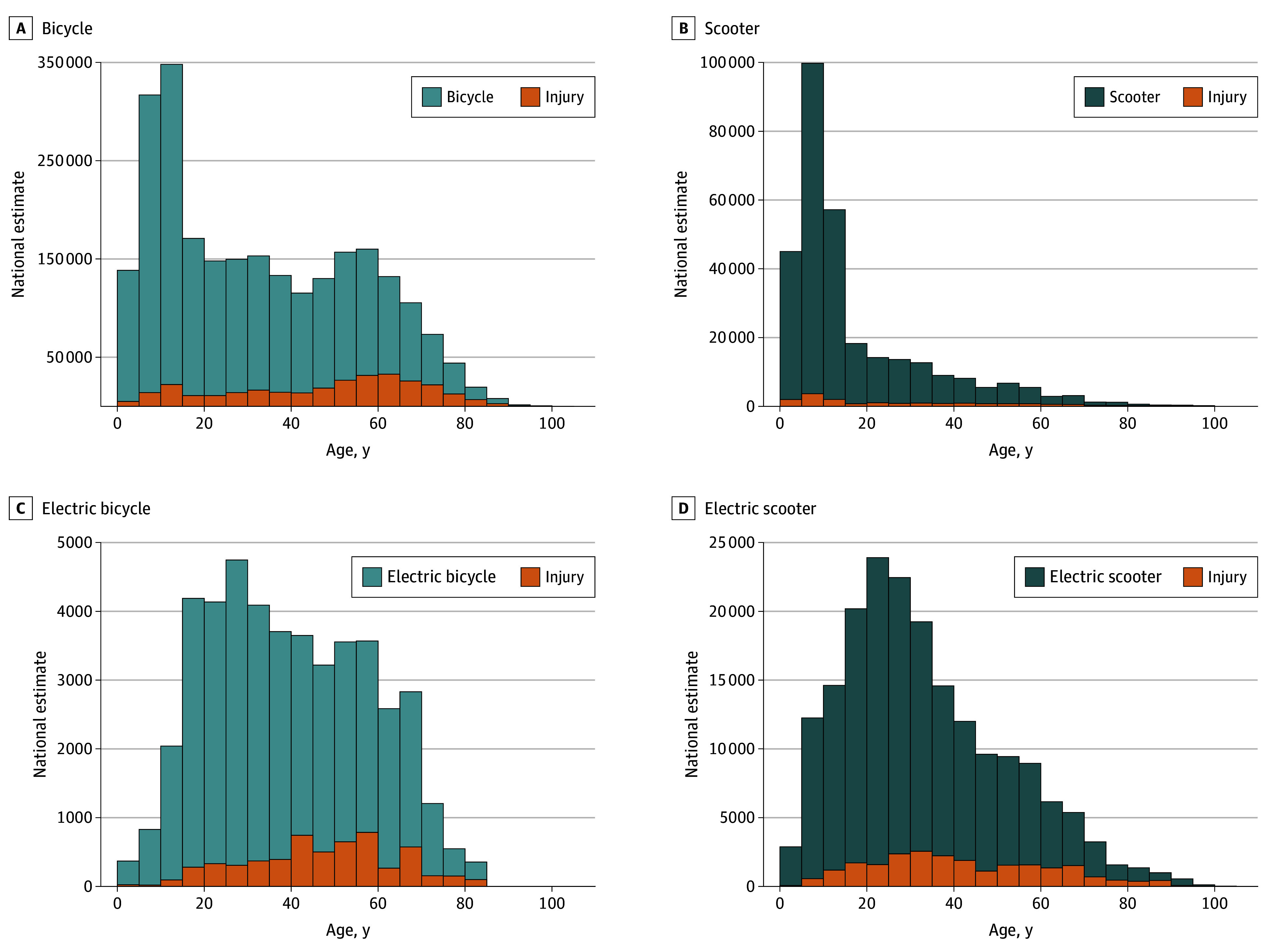
Estimated National Injuries and Hospitalizations by Patient Age and Vehicle Type Injury indicates those resulting in hospitalization.

Age at injury significantly differed across vehicle groups. The median age of injured conventional bicycle riders was 30 (IQR, 13-53) years vs 39 (IQR, 25-55) years for e-bicyclists (*P* < .001). Scooter riders had a median age of 11 (IQR, 7-24) years at the time of injury vs 30 (IQR, 20-45) years for e-scooter riders (*P* < .001) (Table 1 and Figure 3). As a group, those injured from EV accidents were significantly older than those injured from conventional vehicles (age, 31 vs 27 years; *P* < .001) (eTable 1 in Supplement 1). Across all injured riders, the median age rose from 23 (IQR, 11-48) to 32 (IQR, 14-53) years from 2017 to 2022.

**Table 1.** Injury Characteristics by Vehicle Type, 2017-2022

Characteristic	No. (%)				*P* value^a^
	Bicycle	Scooter	e-Bicycle	e-Scooter	
Total	2 499 843 (82)	304 783 (12)	45 586 (2)	189 517 (7.6)	NA
95% CI	1 948 539-3 051 147	232 466-377 099	17 684-73 488	126 101-252 932	NA
NEISS cases	69 708	10 561	1038	5316	NA
Age, median (IQR), y	30 (13-53)	11 (7-24)	39 (25-55)	30 (20-45)	<.001
Sex					
Female	655 230 (26)	111 778 (37)	11 610 (25)	67 691 (36)	<.001
Male	1 844 472 (74)	193 005 (63)	33 976 (75)	121 738 (64)	
Race and ethnicity					
American Indian	14 154 (0.6)	1752 (0.6)	60 (0.1)	934 (0.5)	.36
Asian	34 581 (1)	5774 (2)	1203 (3)	4373 (3)	.02
Black	273 142 (11)	50 887 (17)	11 147 (24)	47 926 (25)	<.001
Hispanic	118 844 (12)	21 502 (17)	2003 (11)	13 803 (13)	.008
Pacific Islander	3121 (0.1)	517 (0.2)	0	290 (0.2)	.73
White	1 242 238 (50)	139 534 (46)	20 441 (45)	94 101 (50)	.53
Other^b^	76 288 (3)	11 502 (4)	1079 (2)	4677 (3)	.17
Body region injured					
Head or neck	730 178 (29)	92 696 (30)	14 664 (32)	61 378 (32)	.04
Trunk	389 200 (16)	20 075 (6.6)	6764 (15)	19 344 (10)	<.001
Upper extremity	787 594 (32)	109 871 (36)	11 967 (26)	53 532 (28)	<.001
Lower extremity	542 615 (22)	80 140 (26)	11 829 (26)	52 743 (28)	<.001
Diagnosis type					
Blunt injury	722 819 (29)	82 682 (27)	12 219 (27)	53 631 (28)	.16
Sharp injury	358 930 (14)	52 149 (17)	6413 (14)	24 549 (13)	<.001
Orthopedic/fracture	652 861 (26)	88 029 (29)	12 612 (28)	55 534 (29)	.004
Burn/shock	1242 (<0.1)	64 (<0.1)	99 (0.2)	269 (0.1)	.01
Neurologic	64 505 (3)	5804 (2)	380 (0.8)	4423 (2)	.009
Internal	275 450 (11)	27 264 (9)	6134 (13)	21 499 (11)	.003
Dental/facial	23 565 (0.9)	4457 (1.5)	40 (<0.1)	1716 (0.9)	<.001
Amputation/avulsion	14 465 (0.6)	1713 (0.6)	339 (0.7)	769 (0.4)	.64
Other	103 776 (4)	12 039 (4)	858 (2)	7177 (4)	.07
Unknown injury	365 863 (15)	41 796 (14)	7195 (16)	26 383 (14)	.61
Alcohol involved	65 858 (4)	7235 (3)	3034 (7)	14 869 (9)	<.001
Drugs involved	27 103 (2)	2088 (1)	1148 (3)	3045 (2)	.02
Helmet used	265 896 (53)	14 717 (41)	6095 (44)	18 625 (43)	.001
Hospital type					
Urban	1 778 847 (71)	213 182 (70)	36 726 (81)	159 265 (84)	.03
Rural	645 500 (26)	70 527 (23)	8601 (19)	26 898 (14)	.04
Children’s	75 496 (3)	21 074 (7)	260 (0.6)	3354 (2)	<.001
Admitted	301 213 (12)	17 677 (5.8)	5703 (13)	23 251 (12)	<.001
Death	3002 (0.1)	94 (<0.1)	17 (<0.1)	319 (0.2)	.11

Abbreviations: NA, not applicable; NEISS, National Electronic Injury Surveillance System.

^a^ Wilcoxon rank-sum test for complex survey samples; χ^2^ test with Rao and Scott second-order correction.

^b^ Includes individuals identified in the NEISS database as African, Bengali, Central American, Dominican, Guatemalan, Honduran, Lithuanian, Mexican, Multiracial, Nepali, Puerto Rican, Russian, Somali, South American, Spanish, Turkish, and Ukrainian.

#### Race and Ethnicity

There were no significant differences in the percentage of White individuals injured in each vehicle group (Table 1). Black individuals made up 11% of the injured conventional bicyclist cohort and 24% of the e-bicyclist cohort (*P* = .11), and 17% of the conventional scooter cohort and 25% of the e-scooter cohort (*P* < .001) (Table 1). Overall, 25% of EV injuries occurred in Black individuals compared with 12% of non-EV injuries (*P* < .001) (eTable 2 in Supplement 1).

#### Hospital Location and Injury Type

Electric vs conventional vehicle injuries were significantly more common in urban areas (83% vs 71%; *P* = .008) and less common in rural areas (15% vs 26%; *P* = .02) (eTable 1 in Supplement 1). Head/neck, orthopedic, and neurologic injury rates did not statistically differ when comparing injuries from bicycles with e-bicycles or scooters with e-scooters (Table 1). e-Scooter riders were more likely to sustain internal injuries than conventional scooter riders (11% vs 9%; *P* = .002), and upper extremity injuries were more common among conventional riders (32% vs 28%; *P* < .001) (Table 1).

### Safe Riding Choices

#### Helmets and Alcohol

At the time of injury, 53% of bicyclists and 44% of e-bicyclists wore helmets (*P* = .12). The EV riders overall were significantly less likely to wear a helmet at the time of injury (43% vs 52%; *P* = .02) (eTable 1 in Supplement 1). Comparing injured men and women across all vehicle types, men were less likely to wear helmets (50% vs 55%; *P* = .004). Alcohol involvement during injury was more common among e-bicyclists (7%) than conventional bicyclists (4%) (*P* < .001) and among e-scooter (9%) than scooter riders (3%) (*P* < .001).

### Associations With Hospitalization

Adjusting for age, Black riders across all vehicle groups had 24% lower odds of hospitalization compared with White riders (adjusted odds ratio [AOR], 0.76; 95% CI, 0.59-0.98; *P* = .04) (Table 2). Head injury (AOR, 1.20; 95% CI, 1.03-1.41), drug use (AOR, 2.70; 95% CI, 2.26-3.23), and alcohol use (AOR, 1.71; 95% CI, 1.37-2.13) at the time of vehicle injury were each associated with hospitalization among all vehicle types (Table 2). There was no association between helmet use at the time of injury and hospitalization (Table 2).

**Table 2.** Adjusted ORs for Hospitalization by Micromobility Vehicle Group

Characteristic	Adjusted OR (95% CI)^a^						
	Bicycle (n = 2 499 843)	Scooter (n = 304 783)	e-Bicycle (n = 45 586)	e-Scooter (n = 189 517)	Conventional vehicle (n = 2 804 626)	e-Vehicle (n = 235 103)	All vehicles (n = 3 039 728)
Race^b^							
Black	0.72 (0.55-0.93)	1.06 (0.66-1.70)	0.49 (0.25-0.97)	0.91 (0.70-1.19)	0.74 (0.57-0.97)	0.82 (0.60-1.14)	0.76 (0.59-0.98)
White	1 [Reference]	1 [Reference]	1 [Reference]	1 [Reference]	1 [Reference]	1 [Reference]	1 [Reference]
Other	1.07 (0.86-1.34)	0.96 (0.52-1.78)	1.56 (0.89-2.74)	1.68 (1.00-2.82)	1.07 (0.87-1.31)	1.66 (1.06-2.60)	1.11 (0.91-1.35)
Unknown	0.93 (0.67-1.28)	0.88 (0.63-1.22)	0.91 (0.42-1.98)	0.86 (0.62-1.19)	0.92 (0.67-1.27)	0.87 (0.61-1.24)	0.92 (0.68-1.26)
Helmet used	0.95 (0.83-1.08)	0.91 (0.66-1.25)	0.95 (0.53-1.70)	0.98 (0.55-1.74)	0.94 (0.83-1.07)	0.98 (0.58-1.65)	0.95 (0.82-1.09)
Head injury	1.22 (1.02-1.45)	1.29 (1.02-1.63)	1.86 (1.25-2.76)	0.86 (0.64-1.14)	1.22 (1.03-1.45)	1.01 (0.78-1.30)	1.20 (1.03-1.41)
Drug	2.80 (2.19-3.59)	2.68 (1.26-5.69)	2.46 (1.01-6.01)	2.07 (1.02-4.18)	2.80 (2.23-3.52)	2.15 (1.31-3.52)	2.70 (2.26-3.23)
Alcohol	1.59 (1.29-1.95)	3.24 (1.61-6.49)	1.57 (0.86-2.85)	1.84 (1.26-2.70)	1.68 (1.34-2.11)	1.81 (1.32-2.47)	1.71 (1.37-2.13)
Injury occurred on a street	1.44 (1.26-1.64)	1.78 (1.22-2.60)	0.66 (0.43-1.02)	1.31 (1.02-1.68)	1.47 (1.30-1.66)	1.16 (0.93-1.43)	1.45 (1.29-1.62)

Abbreviation: OR, odds ratio.

^a^ All covariates adjusted for age.

^b^ Results for only Black, White, other, and unknown are provided because these are the races identified in the National Electronic Injury Surveillance System codebook.

## Discussion

This cross-sectional study suggests a substantial increase in the number of injuries and hospitalizations from e-scooters and e-bicycles from 2017 to 2022. During this interval, injuries from conventional scooters and bicycles were stable; however, hospitalization rates among conventional and micromobility vehicles rose. Therefore, recent growth in health care use across the micromobility market appears to be accounted for by both an increase in EV injuries and conventional vehicle hospitalizations.

The increase in EV injuries and all micromobility-associated hospitalizations from 2017 to 2022 most likely reflects the growing popularity of these vehicles. Shareable micromobility platforms have expanded greatly across the US over this time period, increasing micromobility availability in cities.^5^ In addition to accessibility, these vehicles have become popular consumer options because they are an affordable, environmentally conscious, and enjoyable transportation option that allows for exercise.^8^ The electric micromobility industry may continue to grow in popularity, as rollout of electric transportation options, including e-bicycles and e-scooters, was outlined as a top priority to mitigate climate change according to the 2021 Glasgow Summit.^17,18,19^ This is especially true in the US, as Congress is actively considering approval of subsidies for electric bicycle purchases.^20^

As the popularity of micromobility, and especially electric micromobility, grows, it is important to consider the differences between conventional and electric micromobility ridership. Injured riders of electric-powered bicycles and scooters are older and more likely to be Black than those of conventional micromobility vehicles. The trend of increasing hospitalizations despite stable rates of combined injuries may be linked to demographic shifts, with the median age of injured riders increasing by nearly a decade across our study period. The NEISS data also suggest that EV injuries are more common in urban environments, a pattern that mimics the faster adoption of electric automobiles by US cities than towns.^21^ In addition, injured EV riders are more likely to participate in risky behaviors, such as intoxicated and helmetless riding, than conventional vehicle users.

The technological advantages of electric micromobility vehicles may account in part for their appeal to older adults. In e-bicycling, electric pedal assistance allows riders to accelerate quickly, often up to 28 miles per hour, allowing users to cover more distance with less effort and time.^9^ e-Bicycles have lowered barriers to cycling for older adults, a group at risk for physical inactivity.^9,10^ Biking has clear-cut physical and cognitive health benefits for older adults, so this extension of biking accessibility to older e-bicyclists should be considered a boon of the new technology.^22,23^ However, as injured e-bicycle riders are older than conventional bicyclists, the unique safety considerations for older cyclists should be a focus of ongoing study.

To our knowledge, the finding of increased frequency of injury among Black EV riders is novel to this study, consistent in both the e-scooter vs conventional scooter and e-bicycle vs conventional bicycle cohorts (eTable 1 in Supplement 1), and the explanation is not clear from this dataset. The observed lower odds of hospitalization for Black compared with White riders, even after adjusting for age, may signal potential disparities. However, the NEISS database does not provide detailed clinical narratives for each encounter that would allow for definitive conclusions about the reasons for these differences. As such, while our findings raise questions that are aligned with concerns about disparities in health care access and quality, including the possible influence of systemic issues, they should be interpreted with caution.^24,25^ Further detailed studies are necessary to understand the underlying factors associated with these findings.

Helmet use is less common overall in injured EV users compared with non-EV users, although this difference was not statistically significant when comparing e-scooter with conventional scooter or e-bicycle with conventional bicycle groups. Although helmet use varies significantly across the globe, Swiss studies have identified helmet use in up to 69% of e-bicyclists, compared with 41% to 53% among users of micromobility vehicles in the present study.^26^ In contrast, Scandinavian studies reveal much lower helmet use among e-scooter riders, with only 2.1% wearing helmets, and a higher rate of intoxication at 39.5%, compared with 9% in our study.^27^ These discrepancies suggest that e-scooter riders may engage in riskier behaviors, although further study is needed for direct comparisons.

The low helmet use reported herein may be related to challenges with helmet rental in bicycle and scooter sharing platforms, which do not consistently offer helmet rental. Although helmet use was not associated with lower hospitalization odds, head injury was associated with greater odds of hospitalization, and helmet use is perhaps the best protective mechanism to prevent brain injury while bicycling.^28^ Therefore, helmet use during micromobility use should be highly encouraged. Additionally, our findings suggest that helmets are worn less frequently by men across micromobility vehicle groups, similar to findings in bicyclists in the US and in motorcyclists in India.^29,30,31^ While public health campaigns should broadly target all users, specific strategies might be needed for subgroups that are less likely to wear helmets.

In addition to helmet safety, our study points to broader implications for public health strategies that warrant further investigation. Developing micromobility-friendly urban infrastructure may enhance safety for riders. A study in New York City reported that nearly a quarter of micromobility users encountered bike lane obstructions, often diverting into vehicle traffic lanes.^32^ This highlights the need for robust and better integrated bike lanes that are suitable for micromobility riders, minimizing interactions with pedestrians and motor vehicles. The enforcement of mandatory helmet laws and speed limits for EVs is also essential, as speed restrictions may reduce the number of injuries.^33^ Such regulations, alongside vigorous public education campaigns, could substantially boost helmet use and promote safe riding practices. Additionally, targeted interventions in high-use areas could decrease injury incidences by addressing local population needs and behaviors.

### Limitations

Many limitations of the present study are intrinsic to the NEISS database, such as the lack of complete clinical information with each injury report. Helmet use data, for example, were not included in each clinical narrative, so conclusions drawn here may be limited. However, given the incidental nature of helmet use mentions in NEISS narratives, we believe that our findings on helmet use are likely free of selection bias. Additionally, the database lacks specific exposure data, such as frequency and context of EV use (eg, time of day injuries occurred), which could influence injury outcomes. Moreover, the EDs sampled in the NEISS database may not entirely reflect the health care system across the US, although NEISS projections have been estimated to be 89% to 98% accurate.^6,34,35^ Finally, the current study may underestimate rates of injury, as some patients may be reluctant to seek medical care for injuries or may be seen for injuries in non-ED environments.

## Conclusions

As the popularity of micromobility vehicles increases, the findings of this cross-sectional study suggest that injuries and hospitalizations are increasing among US riders of small EVs. The population of injured EV riders is distinct from that of individuals using conventional bicycles and scooters. As US cities work to adapt to innovations in a rapidly changing transportation sector, there is an opportunity to institute national change in educational policies, infrastructure, and law to recenter on safety. Academic priorities should include understanding the low hospitalization rates among Black individuals injured in micromobility accidents and the underpinnings of low helmet usage in men and EV riders.

## Notes

Supplement 1. **eTable 1.** Estimated National Injuries and Hospitalizations per Year **eTable 2.** Injury Characteristics by Electric vs Non-electric Vehicle Type **eTable 3.** Hospitalization Characteristics by Vehicle Type

Supplement 2.
Data Sharing Statement
