# Errors in Table 1

**DOI:** 10.1001/jamanetworkopen.2025.60443

**Published:** 2026-02-03

In the Original Investigation titled “Injuries With Electric vs Conventional Scooters and Bicycles,”^1^ published July 23, 2024, there were 2 numeric errors in Table 1. The 95% CI listed for the Bicycle column as “948 539” should have been “1 948 539.” The total number and percentage value for the e-Scooter column given as “67 691 (36)” should have been “189 517 (7.6).” No other values are affected by these changes. This article has been corrected.^1^
