# Supplementary material for: Injuries With Electric vs Conventional Scooters and Bicycles
Source: JAMA Netw Open. 2024 Jul 23;7(7):e2424131. doi: 10.1001/jamanetworkopen.2024.24131 (PMC11267411; doi:10.1001/jamanetworkopen.2024.24131)
Supplement: Supplement 1. — eTable 1. Estimated National Injuries and Hospitalizations per Year eTable 2. Injury Characteristics by Electric vs Non-electric Vehicle Type eTable 3. Hospitalization Characteristics by Vehicle Type [file jamanetwopen-e2424131-s001.pdf]

Supplementary Online Content

Fernandez A, Li KD, Patel HV, et al. Injuries with electric vs conventional scooters and bicycles. *JAMA Netw Open*. 2024;7(7):e2424131. doi:10.1001/jamanetworkopen.2024.24131

eTable 1. Estimated National Injuries and Hospitalizations per Year

eTable 2. Injury Characteristics by Electric vs Non-electric Vehicle Type

eTable 3. Hospitalization Characteristics by Vehicle Type

This supplementary material has been provided by the authors to give readers additional information about their work.

© 2024 Fernandez A et al. *JAMA Network Open.*

eTable 1. Estimated National Injuries and Hospitalizations per Year (95% Confidence Interval)

	2017	2018	2019	2020	2021	2022	p-value[1]
			**Injuries**				
**All**	514,382 (412,179-616,584)	479,629 (371,358-587,900)	493,364 (383,464-603,265)	504,560 (394,086-615,035)	496,391 (383,257-609,525)	551,402 (414,827-687,978)	0.28
**Bicycles**	456,466 (362,994-549,939)	423,154 (324,357-521,952)	416,547 (319,533-513,562)	425,419 (327,498-523,340)	374,692 (286,286-463,097)	403,565 (299,036-508,094)	0.06
**Scooters**	48,598 (39,080-58,116)	39,411 (28,904-49,918)	45,277 (31,922-58,633)	44,492 (34,033-54,950)	59,508 (40,070-78,945)	67,497 (44,376-90,618)	0.08
**eScooters**	8,566 (5,522-11,611)	15,449 (7,998-22,899)	29,325 (14,659-43,990)	29,023 (14,376-43,669)	50,307 (32,394-68,219)	56,847 (39,673-74,022)	**0.002**
**eBicycles**	751 (0-1,586)	1,615 (318-2,912)	2,215 (614-3,815)	5,627 (1,107-10,147)	11,885 (3,869-19,900)	23,493 (11,043-35,944)	**<0.001**
			**Hospitalizations**				
**All**	48,660 (32,572-64,749)	51,292 (33,321-69,262)	53,903 (37,318-70,489)	64,263 (45,703-82,823)	60,039 (43,016-77,062)	69,686 (40,530-98,842)	**0.002**
**Bicycles**	46,127 (30,347-61,907)	47,703 (30,016-65,390)	48,397 (32,671-64,123)	55,907 (38,588-73,227)	48,141 (32,597-63,685)	54,937 (29,378-80,496)	**0.003**
**Scooters**	1,677 (1,167-2,186)	1,770 (1,018-2,522)	1,648 (941-2,356)	3,149 (1,940-4,358)	4,122 (2,085-6,160)	5,310 (2,460-8,159)	**<0.001**
**eScooters**	791 (219-1,363)	1,583 (765-2,401)	3,670 (1,450-5,891)	4,633 (2,424-6,842)	6,257 (3,685-8,828)	6,317 (3,584-9,050)	0.96
**eBicycles**	66 (0-139)	235 (0-520)	188 (0-385)	574 (0-1,154)	1,519 (321-2,716)	3,122 (1,678-4,567)	0.54

[1]Injury p-values were derived from linear regression on log-counts, while hospitalization p-values were obtained from logistic regression on hospitalization odds with year as a covariate.

© 2024 Fernandez A et al. *JAMA Network Open.*

eTable 2. Injury Characteristics by Electric vs Non-electric Vehicle Type

Characteristic	**Non-electric**, N = 2,804,626 (92.3%)	**Electric**, N = 235,103 (7.7%)	p-value[1]
**Age**, Median (IQR)	27 (12, 51)	31 (21, 48)	**<0.001**
**Male Sex** (%)	2,037,476 (73)	155,714 (66)	**0.05**
**Race** (%)			
Asian	40,356 (1.4)	5,576 (2.4)	**0.03**
Black	324,029 (12)	59,074 (25)	**<0.001**
Native American	15,906 (0.6)	994 (0.4)	0.32
Pacific Islander	3,638 (0.1)	290 (0.1)	0.86
White	1,381,772 (49)	114,542 (49)	0.91
Other[2]	87,791 (3.1)	5,756 (2.4)	0.24
**Ethnicity Hispanic or Latino** (%)	140,345 (12)	15,806 (12)	>0.9
**Body Region Injured** (%)			
Head or Neck Injury	822,874 (29)	76,043 (32)	**0.01**
Trunk Injury	409,275 (15)	26,108 (11)	**0.002**
Upper Extremity Injury	897,465 (32)	65,499 (28)	**<0.001**
Lower Extremity Injury	622,756 (22)	64,572 (27)	**<0.001**
**Diagnosis Type** (%)			
Blunt Injury	805,501 (29)	65,849 (28)	0.4
Sharp Injury	411,079 (15)	30,962 (13)	**0.008**
Orthopedic/Fracture	740,889 (26)	68,146 (29)	**0.01**
Burn/Shock	1,307 (<0.1)	368 (0.2)	**0.008**
Neurological Injury	70,309 (2.5)	4,803 (2.0)	0.2
Internal Injury	302,713 (11)	27,634 (12)	**0.03**
Dental/Facial	28,022 (1.0)	1,756 (0.7)	0.10
Amputation/Avulsion	16,178 (0.6)	1,108 (0.5)	0.41
Other Injury	115,815 (4.1)	8,035 (3.4)	0.09
Unknown Injury	407,659 (15)	33,577 (14)	0.84
**Alcohol Involved** (%)[3]	73,093 (4.0)	17,902 (8.6)	**<0.001**
**Drug(s) Involved** (%)[3]	29,191 (1.6)	4,193 (2.0)	0.09
**Helmet Used** (%)	280,613 (52)	24,720 (43)	**0.02**
**Hospital Type** (%)			
Urban	1,992,029 (71)	195,990 (83)	**0.008**
Rural	716,027 (26)	35,499 (15)	**0.02**
Children's	96,570 (3.4)	3,613 (1.5)	**<0.001**
**Left Without Being Seen** (%)	65,951 (2.4)	6,955 (3.0)	0.16
**Treated and Released** (%)	2,416,600 (86)	198,858 (85)	0.08
**Admitted** (%)	318,889 (11)	28,954 (12)	0.18
Treated and Transferred	41,323 (1.5)	3,350 (1.4)	0.86
Treated and Hospitalized	261,490 (9.3)	23,880 (10)	0.27
Held for Observation	16,076 (0.6)	1,724 (0.7)	0.35
**Death** (%)	3,097 (0.1)	336 (0.1)	0.58

[1]Wilcoxon rank-sum test for complex survey samples; chi-squared test with Rao & Scott's second-order correction.
[2]Includes individuals identified as African, Bengali, Multiracial, Central American, Dominican, Guatemalan, Honduran, Lithuanian, Mexican, Nepali, Puerto Rican, Russian, Somali, South American, Spanish, Turkish, and Ukrainian.
[3]Information on drug and alcohol use was only available in the dataset starting from 2019.

© 2024 Fernandez A et al. *JAMA Network Open.*

eTable 3. Hospitalization Characteristics by Vehicle Type

**Characteristic**	**Bicycle**, N = 301,213 (87%)	**Scooter**, N = 17,677 (5.1%)	**eBicycle**, N = 5,703 (1.6%)	**eScooter**, N = 23,251 (6.7%)	**p-value**[1]
**Age,** median (IQR)	52 (30, 65)	22 (9, 47)	49 (37, 57)	39 (27, 57)	**<0.001**
**Male Sex** (%)	237,772 (79)	12,556 (71)	4,519 (79)	17,029 (73)	**0.05**
**Race** (%)					
Asian	4,003 (1.3)	433 (2.5)	142 (2.5)	526 (2.3)	**0.05**
Black	23,093 (7.7)	3,775 (21)	772 (14)	5,414 (23)	**<0.001**
Native American	1,095 (0.4)	178 (1.0)	0 (0)	288 (1.2)	0.15
Pacific Islander	370 (0.1)	16 (<0.1)	0 (0)	0 (0)	0.90
White	153,816 (51)	8,477 (48)	3,059 (54)	12,421 (53)	0.77
Other[2]	8,413 (2.8)	413 (2.3)	194 (3.4)	743 (3.2)	0.85
**Ethnicity Hispanic or Latino** (%)	12,138 (9.6%)	1,159 (13%)	201 (8.0%)	2,088 (14%)	**0.03**
**Body Region Injured** (%)					
Head or Neck Injury	95,819 (32)	5,833 (33)	2,610 (46)	6,860 (30)	0.07
Trunk Injury	101,665 (34)	3,201 (18)	1,108 (19)	5,144 (22)	**<0.001**
Upper Extremity Injury	44,527 (15)	3,380 (19)	641 (11)	2,817 (12)	0.11
Lower Extremity Injury	49,622 (16)	4,994 (28)	1,306 (23)	8,298 (36)	**<0.001**
**Diagnosis Type** (%)					
Blunt Injury	16,435 (5.5)	619 (3.5)	251 (4.4)	1,328 (5.7)	0.41
Sharp Injury	13,399 (4.4)	1,160 (6.6)	413 (7.2)	876 (3.8)	0.15
Orthopedic/Fracture	152,824 (51)	9,956 (56)	2,957 (52)	13,754 (59)	**0.001**
Burn/Shock	79 (<0.1)	16 (<0.1)	0 (0)	17 (<0.1)	0.56
Neurological Injury	7,581 (2.5)	253 (1.4)	34 (0.6)	363 (1.6)	0.06
Internal Injury	67,048 (22)	3,507 (20)	1,658 (29)	5,029 (22)	0.45
Dental/Facial	1,010 (0.3)	256 (1.4)	0 (0)	16 (<0.1)	**0.03**
Amputation/Avulsion	1,068 (0.4)	0 (0)	0 (0)	23 (0.1)	0.62
Other Injury	9,739 (3.2)	524 (3.0)	34 (0.6)	420 (1.8)	0.08
Unknown Injury	39,299 (13)	1,751 (9.9)	325 (5.7)	1,823 (7.8)	0.10
**Alcohol Involved** (%)[3]	14,751 (7.1)	1,814 (13)	548 (10)	3,065 (15)	**0.005**
**Drug(s) Involved** (%)[3]	8,686 (4.2)	575 (4.0)	320 (5.9)	806 (3.9)	0.82
**Helmet Used** (%)	49,753 (57)	1,753 (42)	1,122 (47)	3,327 (47)	0.06
**Hospital Type** (%)					
Urban	250,493 (83)	14,008 (79)	4,956 (87)	21,262 (91)	0.21
Rural	44,139 (15)	2,357 (13)	701 (12)	1,633 (7.0)	0.25
Children's	6,581 (2.2)	1,311 (7.4)	47 (0.8)	356 (1.5)	**<0.001**
**Treated and Transferred**	37,576 (12)	3,747 (21)	416 (7.3)	2,934 (13)	**0.03**
**Treated and Hospitalized**	248,195 (82)	13,295 (75)	5,029 (88)	18,851 (81)	0.15
**Held for Observation**	15,442 (5.1)	634 (3.6)	258 (4.5)	1,466 (6.3)	0.54

[1]Wilcoxon rank-sum test for complex survey samples; chi-squared test with Rao & Scott's second-order correction.
[2]Includes individuals identified as African, Bengali, Multiracial, Central American, Dominican, Guatemalan, Honduran, Lithuanian, Mexican, Nepali, Puerto Rican, Russian, Somali, South American, Spanish, Turkish, and Ukrainian.
[3]Information on drug and alcohol use was only available in the dataset starting from 2019.

© 2024 Fernandez A et al. *JAMA Network Open.*
