# Supplementary material for: Injuries With Electric vs Conventional Scooters and Bicycles
Source: JAMA Netw Open. 2024 Jul 23;7(7):e2424131. doi: 10.1001/jamanetworkopen.2024.24131 (PMC11267411; doi:10.1001/jamanetworkopen.2024.24131)
Supplement: Supplement 2. — Data Sharing Statement [file jamanetwopen-e2424131-s002.pdf]

Data Sharing Statement

Fernandez. Injuries With Electric vs Conventional Scooters and Bicycles. *JAMA Netw Open*. Published July 23, 2024. doi:10.1001/jamanetworkopen.2024.24131

Data
Data available: Yes
Data types: Other (please specify)
Additional Information: The data is publicly available through the NEISS database
How to access data: https://www.cpsc.gov/Research--Statistics/NEISS-Injury-Data
When available: With publication

Supporting Documents
Document types: None

Additional Information
Who can access the data: Anyone
Types of analyses: Any
Mechanisms of data availability: Listing of NEISS URL
